# Nutritional status and factors associated with gestational weight gain in the city of São Paulo, 2012 to 2020: a retrospective cohort study

**DOI:** 10.1186/s12884-024-06955-5

**Published:** 2024-11-13

**Authors:** Fernanda Ferreira Corrêa, Eliana de Aquino Bonilha, Wesley Pereira da Silva, Tarcisio Cantos de Melo, Marcus V. L. dos Santos Quaresma, Carmen Simone G. Diniz

**Affiliations:** 1 Departamento dos ciclos da vida, Faculdade de Saúde Pública, Universidade de São Paulo, São Paulo, Brazil; 2 Laboratório de Exercício e Qualidade de Vida, Centro Universitário São Camilo, São Paulo, Brazil

**Keywords:** Nutritional status, Pregnant women, Gestational weight gain, Cohort studies

## Abstract

**Background:**

Gestational weight gain (GWG) is a critical issue related to postpartum health in newborns and mothers. In Brazil, pregnant women’s public health recommends monitoring GWG. Therefore, the objective of this study is to evaluate gestational weight gain and associated health factors of pregnant women monitored at Unified Health System (SUS) in the city of São Paulo between 2012 and 2020.

**Methods:**

This is a retrospective cohort study of pregnant women seen from 2012 to 2020 in São Paulo, Brazil. The database used was from the Integrated Health Care Management System related to the Live Birth Information System. Data distribution was assessed using the Kolmogorov-Smirnov test. Comparisons between groups according to weight gain (LWG vs. AWG vs. EWG) were performed using analysis of variance (ANOVA) with Tukey post hoc. Inclusion criteria considered that pregnant women had a recorded initial weight before 13 weeks and up to 15 days before delivery and a single pregnancy. The final database includes 276.220 pregnant women.

**Results:**

The frequency of women according to initial body mass index (BMI) was 12.004 (4.4%) underweight, 132.049 (48.3%) normal weight, 78.856 (28.8%) overweight, and 50.660 (18.5%) living with obesity. The population consisted of 59.881 (21.9%), 37.217 (13.6%) and 176.471 (64.5%) women with LWG, AWG and EWG, respectively. Weight gain was associated with initial BMI, type of birth, color/ethnicity, marital status, women’s age and antenatal care visits.

**Conclusion:**

The proportion of pregnant women with inadequate weight gain is high, relating initial BMI, type of birth, color/ethnicity, marital status, women’s age and antenatal care visits. Interventions such as nutritional education should be suggested to help achieve adequate GWG.

**Supplementary Information:**

The online version contains supplementary material available at 10.1186/s12884-024-06955-5.

## Introduction

Women’s nutritional status and gestational weight gain (GWG), excessive or insufficient weight gain during pregnancy are critical issues related to postpartum newborn and maternal health [1–3]. Considering nutritional status, body mass index (BMI) is the most monitored parameter used during pregnancy [4]. Previous studies showed that initial BMI is a strong predictor of GWG and that GWG needs to be adjusted according to the category of women’s BMI [4, 5].

In Brazil, pregnant women’s public health recommends monitoring GWG during the gestational period [6]. Brazil has a health service called Unified Health System (SUS), known as SUS, which provides health-related care to the entire Brazilian population free of charge, including people in social vulnerability [7, 8]. The SUS offers prenatal care with several health professionals to monitor and prevent mother and newborn morbidity and mortality [8]. Pregnant women’s GWG reflects maternal physiological adaptations and the growth of the fetus, placenta, and accumulation of amniotic fluid. Thus, GWG is a critical factor to monitor during pregnancy [9] .

Until recently, Atalah et al. [10] charts combined with the 2009 US Institute of Medicine [11] were applied to GWG recommendations. The International Fetal and Newborn Growth Consortium for the 21st Century [12] GWG standards for publication were also used. However, they are limited to women classified as normal weight, and rely on a weight measured between 9 and 14 gestational weeks for GWG calculation, which decreases their utility for monitoring weight gain in the first trimester (the charts start at the 14th gestational week). Kac et al. (2021) [13] developed new GWG charts according to initial BMI for Brazilian women that were adopted into the Brazilian healthcare system. Kac’s charts exclude pregnancies that delivered preterm (< 37 weeks), small (< 10th) or large (> 95th) for gestational age. Delivered infants with low birth weight (weight < 2500 g) or macrosomia (weight > 4000 g) were also excluded. Moreover, the authors included only 10 to 40 weeks of pregnancy to ensure our estimates had reasonable statistical precision.

Despite the increase in research on GWG, there is a notable scarcity of data on the relationship between the nutritional status of pregnant women in the city of São Paulo. As such, we aimed to evaluate gestational weight gain and associated factors of pregnant women monitored at primary care of SUS in the city of São Paulo between 2012 and 2020.

## Methods

### Ethical aspects

This study is part of the research project entitled “Como tornar as intervenções no parto e seus desfechos mais visíveis aos sistemas de informação?” [“How can childbirth interventions and their outcomes be more visible to information systems?”] (project with funding already approved in the Call for Data Science for Maternal and Child Health CNPq - Conselho Nacional de Desenvolvimento Científico e Tecnológico /Bill & Melinda Gates Foundation/2020/2022), approved by the Research Ethics Committee of the Municipal Department of Health of São Paulo, under number 4.829.5.

### Study type and data acquisition

This is a retrospective cohort study from 2012 to 2020 that used data derived from the Primary Health Information System (SIGA) of the city of São Paulo. From the SIGA, we obtained data on pregnant women. Moreover, we linked this dataset to the Live Birth Information System (SINASC). From SINASC, we obtained data about pregnancy and childbirth characteristics.

SIGA database’ quality was evaluated by Corrêa, et al. (2023) [14] in an article about the need of investigating the quality of the collected and recorded data. In this study, the linkage with the SINASC database was carried out, which allowed the analysis of the weight of babies associated with the weight gain of pregnant women.

The probabilistic method was used for the linkage process, considering the probability of 100% of the pairs being true, due to the validation stage. The linkage process completed 5 steps: (I) Treatment of the SIGA base, (II) linkage, (III) validations, (IV) description and (V) calculation of metrics. There are no missing data in the database used, all data is complete, data that presented aberrations were excluded, but this represented less than 1%, considered excellent [15].

From both databases, we extracted raw data (e.g., the maternal weight and height, birth date, type of pregnancy, gestational weeks, type of birth, color/ethnicity, number of antenatal care visits, marital status, and schooling level). From the raw data, we created new variables, including initial weight, final weight, initial gestational age, final gestational age, initial body mass index (BMI), and final BMI. The raw data and transformations used are described in supplementary material 1. Initial BMI was assessed as per WHO recommendations (1998) [16] and weight gain during pregnancy was assessed as per recommendations by Kac et al. (2021) [13]. Kac’s curves used in the present study were developed for adult pregnant women. Their use in adolescent pregnant women has not been tested. However, the Ministry of Health uses these curves for all of Brazil and all age groups of pregnant women, since they are the most appropriate curves for Brazilian women.

### Inclusion and exclusion criteria

Inclusion criteria considered that pregnant women who underwent prenatal care in the City of São Paulo from 2012 to 2020 had a record of initial weight before 13 weeks and up to 15 days before delivery and had a single pregnancy. According to Kac et al. [13], the data included also considered the identification of gestational week and GWG. The exclusion criteria were newborns with congenital anomalies, birth at below 18 or beyond 40 weeks of gestation, incomplete data and extreme data (> six standard deviations).

This study uses data obtained from health services, and therefore, adequate data curation is essential to avoid mistaken estimates. Health professionals who collect anthropometric data are previously trained for the function; however, typing errors and other technical problems lead to incorrect data that was excluded from the analysis.

### Statistical analysis

Data distribution was assessed using the Kolmogorov-Smirnov test. Comparisons between groups according to weight gain (Low Weight Gain - LWG vs. Adequate Weight Gain - AWG vs. Excessive Weight Gain- EWG) were performed using analysis of variance (ANOVA) with Tukey post hoc. In cases of heterogeneous variances, Welch’s correction with Games-Howell post hoc was chosen. Associations between weight gain and descriptive variables were analyzed using the Chi-square test or Fisher’s Exact Test. Finally, multinomial logistic regression models were designed, considering gestational weight gain as the outcome (with AWG as a reference). The independent variables were initial BMI (underweight, overweight, obese vs. normal weight), education (basic 1, basic 2, high school, incomplete college, complete college vs. no education), marital status (single, stable union, divorced and widowed vs. married), color/ethnicity (black, yellow, mixed race, indigenous vs. white), number of health service consultations (1 to 3, 4 to 6, 7 or more vs. none) and age (younger than 15 years old, 15 to 19 years old, 35 to 49 years old and over 49 years old vs. 20 to 34 years old). Multicollinearity was tested and no collinearity was observed between the independent variables. The alpha error adopted to reject the null hypothesis was 5%. Data are presented as mean, standard deviation, odds ratios and the 95% confidence interval. JAMOVI software was used.

## Results

**Fig. 1:**
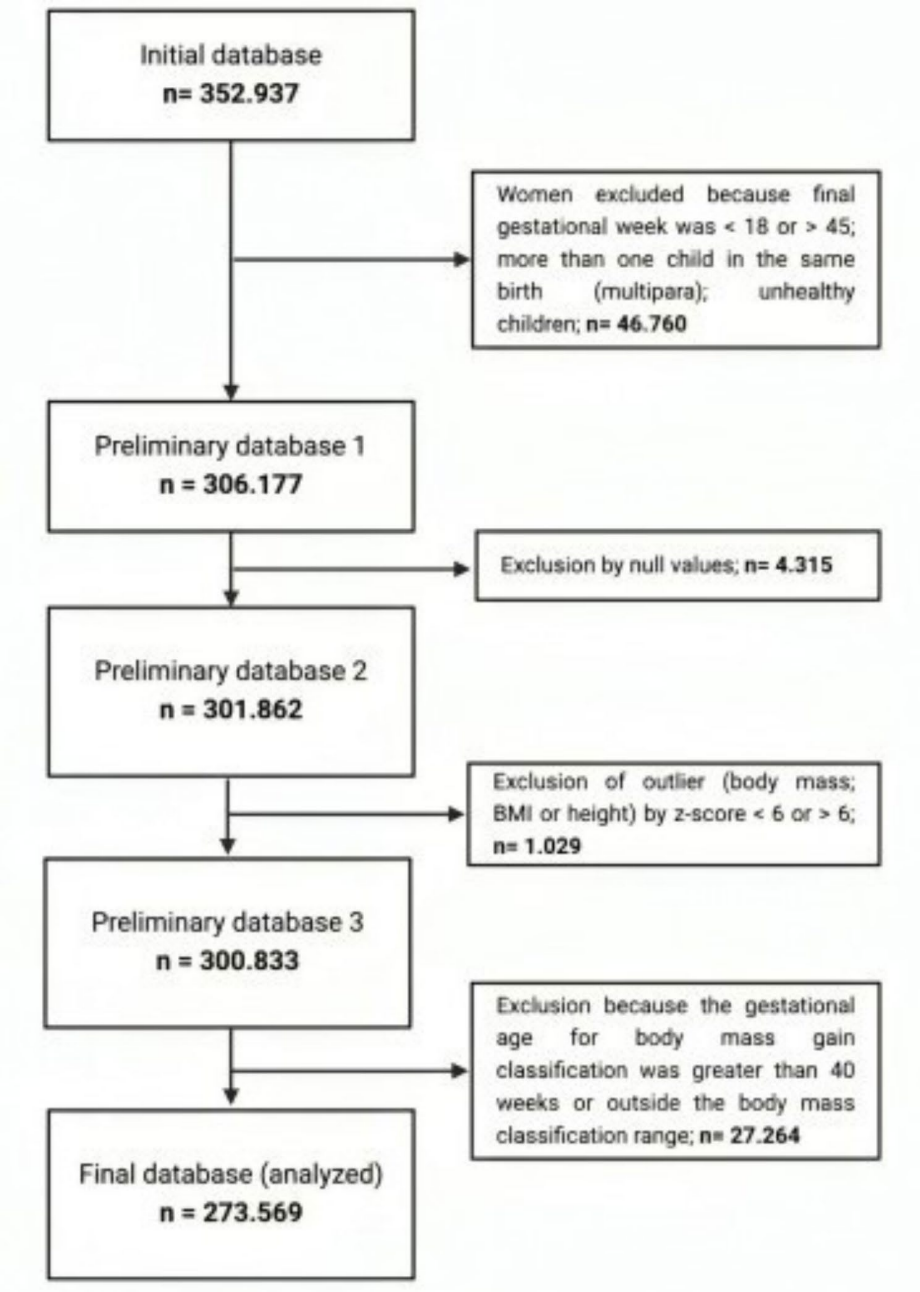
Flowchart of research participants in the present study

The initial database contains 352.937 records. Nevertheless, 46.760 women were excluded because they did not meet the inclusion criteria (final gestational age < 18 or > 40 weeks), were multiparous, or whose children had an inborn error. Then, 4.315 were excluded because they presented incorrect values, and 1.029 were excluded because body mass, BMI or height presented extreme values (< or > than six standard deviations). Furthermore, 24.629 were excluded because their gestational age was < 10 or > 40 weeks, in disagreement with the criteria proposed by Kac et al. Finally, 2635 were excluded because the weight change (variation) did not correspond to the proposal by Kac et al. Figure 1 presents the flowchart of the participants included in the present study.

Table 1 displays the characteristics of women during the gestational period in the city of São Paulo between 2012 and 2020. Welch’s correction was applied for most comparisons of continuous variables. The frequency of women according to BMI was 12.004 (4.4%) underweight, 132.049 (48.3%) normal weight, 78.856 (28.8%) overweight and 50.660 (18.5%) living with obesity. For the analyses, the overweight and obesity groups were combined and referred to as “overweight.”

**Table 1:** Sample characteristics - women in the gestational period between 2012 and 2020 in the city of São Paulo (*n* = 273.569)

Variable	LWG	AWG	EWG	*p*-value
**Age (years) (mean/SD)**	26.7 ± 6.98	25.8 ± 6.86	26.4 ± 6.68	< 0.001
**< 15 years old (n; %)**	1184 (2.0)	890 (2.4)	2.998 (1.7)	< 0.001
**15–19 years old (n; %)**	9.239 (15.4)	7.026 (18.9)	27.345 (15.5)	
**20–34 years old (n; %)**	39.931 (66.7)	24.424 (65.6)	121.974 (69.1)	
**35–49 years old (n; %)**	9.527 (15.9)	4.877 (13.1)	24.153 (13.7)	
**Body weight (kg) (mean/SD)**	68.9 ± 16.50	59.3 ± 8.98	66.2 ± 14.41	< 0.001
**Initial BMI (kg/m** ^**2**^ **) (mean/SD)**	26.8 ± 5.99	23.2 ± 3.02	25.7 ± 5.23	< 0.001
**Initial BMI**				< 0.001
**Underweight (n; %)**	2.922 (4.9)	1.528 (4.1)	7.554 (4.3)	
**Normal weight (n; %)**	24.174 (40.4)	26.177 (70.3)	81.698 (46.3)	
**Overweight (n; %)**	32.784 (54.8)	9.512 (25.6)	87.219 (49.4)	
**Gestational BMI (kg/m** ^**2**^ **) (n; %)**	27.88 ± 5.49	26.46 ± 2.83	30.87 ± 4.91	< 0.001
**Final body weight (kg) (n; %)**	71.61 ± 15.23	67.57 ± 8.55	79.53 ± 13.77	< 0.001
**Δ (Change; kg) (n; %)**	2.69 ± 5.07	8.25 ± 1.31	13.31 ± 4.16	< 0.001
**Initial gestational age (w) (n; %)**	8.63 ± 2.07	8.62 ± 2.07	8.49 ± 2.06	0.020
**Final gestational age (w) (n; %)**	35.09 ± 6.73	37.34 ± 4.55	38.32 ± 3.60	< 0.001
**Type of birth**				< 0.001
**Vaginal delivery (n; %)**	38.477 (64.3)	25.980 (69.8)	107.029 (60.6)	
**Cesarean delivery (n; %)**	21.404 (35.7)	11.237 (30.2)	69.442 (39.4)	
**Marital status**				< 0.001
**Single (n; %)**	31.996 (53.5)	20.909 (56.2)	91.741 (52.0)	
**Married (n; %)**	12.683 (21.2)	6.966 (18.7)	39.632 (22.5)	
**Stable union (marriage not officialized by the government) (n; %)**	14.347 (24.7)	8.830 (23.7)	42.447 (24.1)	
**Divorced (n; %)**	746 (1.2)	454 (1.2)	2.348 (1.3)	
**Widow (n; %)**	89 (0.1)	46 (0.1)	244 (0.1)	
**Schooling level**				< 0.001
**Without (n; %)**	96 (0.2)	46 (0.1)	173 (0.1)	
**Basic studies 1 (n; %)**	1704 (2.8)	864 (2.3)	3.676 (2.1)	
**Basic studies 2 (n; %)**	13.865 (23.2)	8.149 (21.9)	34.390 (19.5)	
**High school (n; %)**	38.393 (64.1)	24.860 (66.8)	119.706 (67.9)	
**Incomplete college (n; %)**	2.531 (4.2)	1.467 (3.9)	8.233 (4.7)	
**Complete college (n; %)**	3.280 (5.5)	1.818 (4.9)	10.242 (5.8)	
**Color/ethnicity**				< 0.001
**White (n; %)**	21.495 (35.9)	13.631 (36.6)	64.263 (36.4)	
**Black (n; %)**	6.020 (10.1)	3.277 (8.8)	15.897 (9.0)	
**Yellow (n; %)**	232 (0.4)	222 (0.6)	783 (0.4)	
**Brown (n; %)**	31.958 (53.4)	19.972 (53.7)	95.103 (53.9)	
**Indigenous (n; %)**	169 (0.3)	111 (0.3)	413 (0.2)	
**Antenatal care visits (n; %)**				< 0.001
**0**	77 (0.1)	31 (0.1)	104 (0.1)	
**1–3**	1.942 (3.2)	623 (1.7)	1.711 (1.0)	
**4–6**	10.292 (17.2)	5.383 (14.5)	16.609 (9.4)	
**≥ 7**	47.538 (79.4)	31.172 (83.8)	157.995 (89.6)	

Legend: kg: kilograms; kg/m^2^: kilograms per square meter; n = number of observations in absolute values; % proportion of the number of observations; Δ difference between the final and initial (change); w: weeks. Analysis of variance (ANOVA) with Tukey post hoc. In cases of heterogeneous variances, Welch correction with Games-Howell post hoc was chosen. Associations between weight gain and descriptive variables were analyzed using the Chi-square test or Fisher’s Exact Test. The alpha error adopted to reject the null hypothesis was 5%

**Fig. 2:**
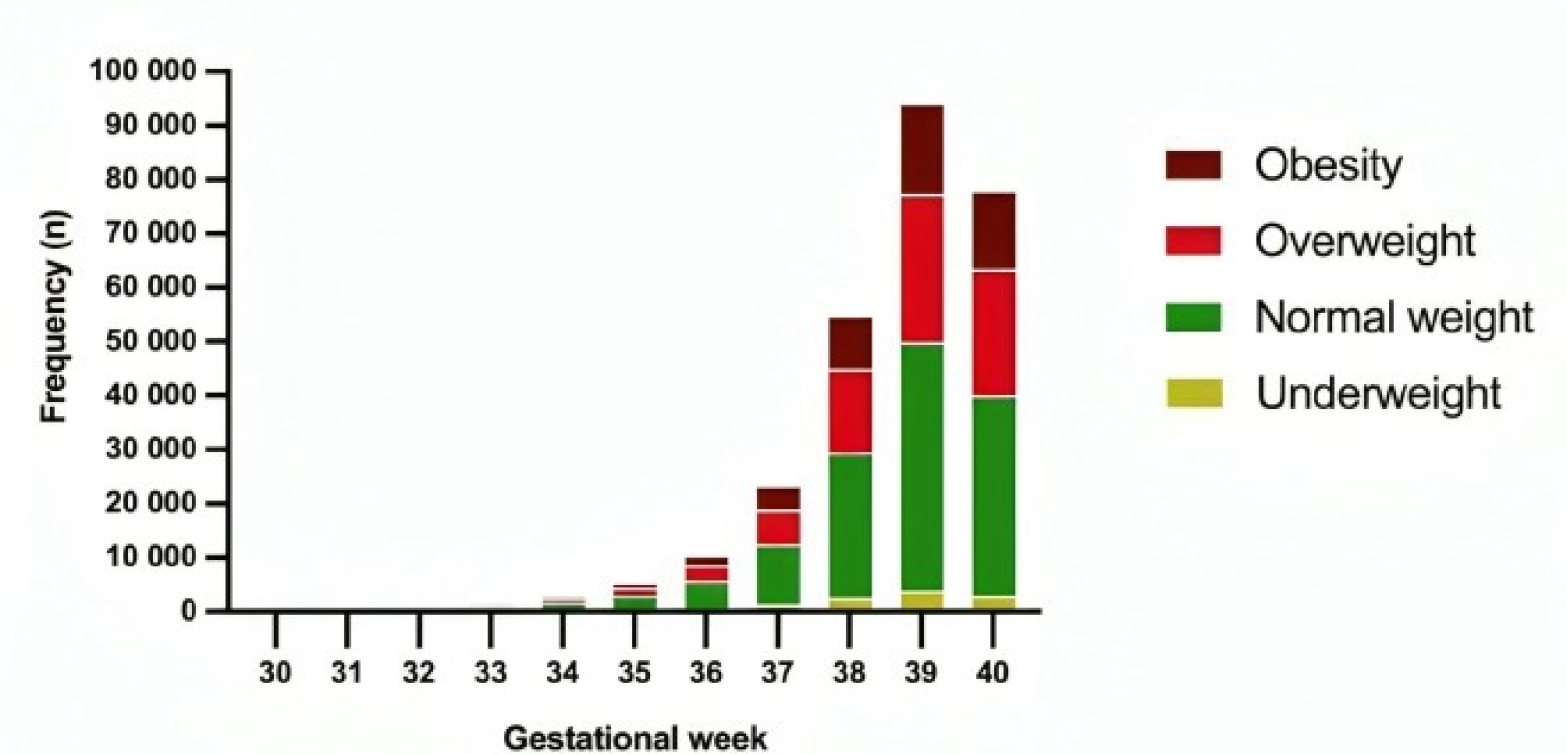
Distribution of women according to initial weight according to WHO, 1998 criteria, São Paulo, 2012 to 2020

**Fig. 3:**
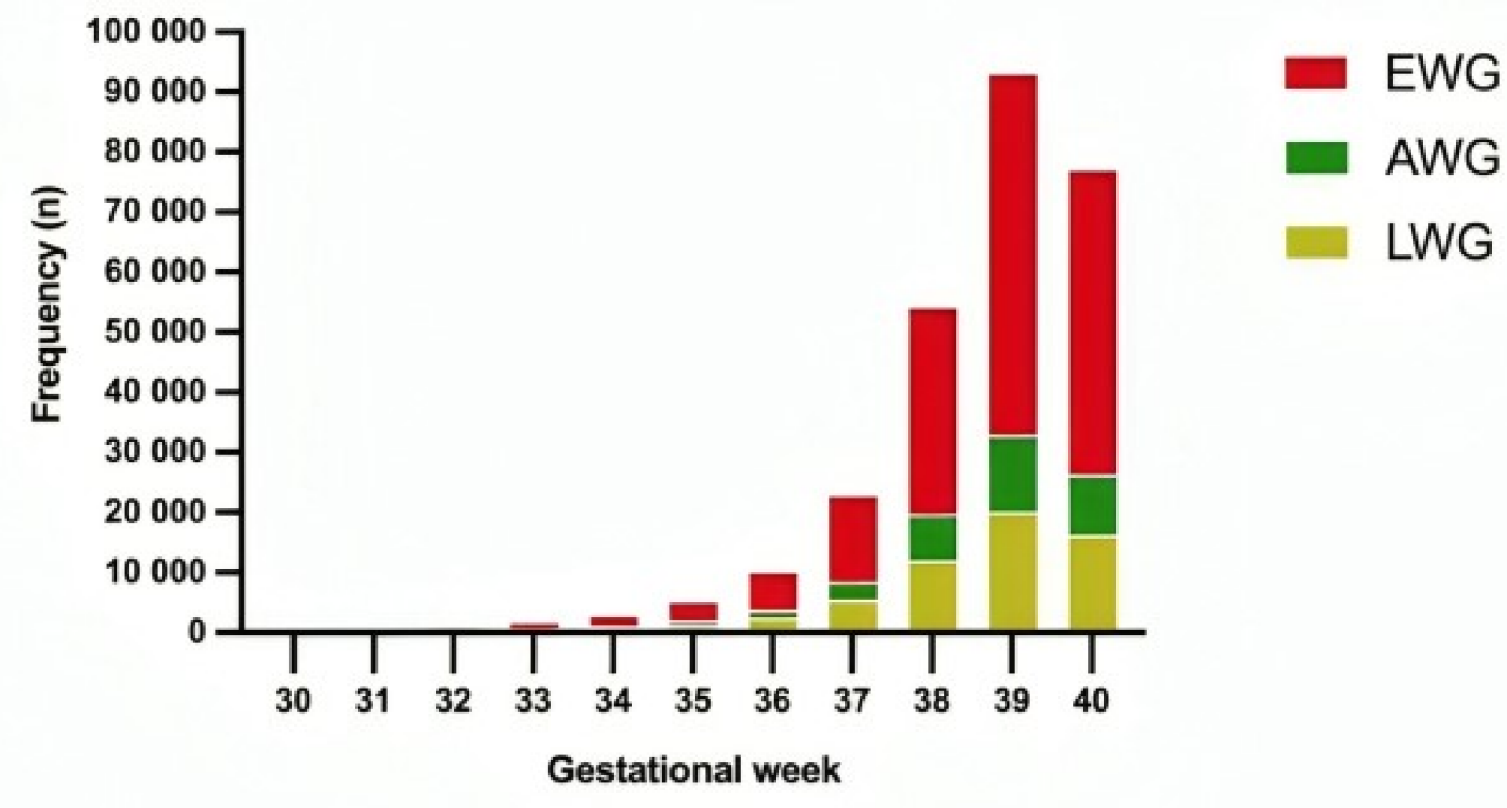
Distribution of pregnant women according to gestational age at birth weight gain during pregnancy according to Kac (2021) criteria, São Paulo, 2012 to 2020

Figure 2 presents the frequency of pregnant women according to gestational age at birth and BMI. The sample consisted of 59.881 (21.9%), 37.217 (13.6%) and 176.471 (64.5%) women with LWG, AWG and EWG, respectively, according to Kac et al. (2021) [13]. Figure 3 shows the distribution of women according to initial weight, WHO, (1998) criteria (World Health Organization, 1998).

### Age

Age (W_(84.853, 2)_ = 226; *p* < 0.001) differed between groups. Women in the AWG group are younger than LWG (MD: -0.96; *p* < 0.001) and EWG (MD: -0.59; *p* < 0.001) groups, while the LWG women group are older than the EWG group (MD: 0.37; *p* < 0.001).

### Body weight and BMI

The initial (W_(100.717, 2)_ = 9613; *p* < 0.001) and gestational (W_(101.257, 2)_ = 25076; *p* < 0.001) body weight differed between the groups. For the initial weight, the AWG group showed lower body weight than LWG (MD: -9.61 kg; *p* < 0.001) and EWG (MD: -6.91 kg; *p* < 0.001) groups. Still, the LWG group showed higher body weight than the EWG group (MD: 2.70 kg; *p* < 0.001). For gestational weight, the AWG group showed lower body weight than the LWG (MD: -4.05 kg; *p* < 0.001) and the EWG (MD: -11.97 kg; *p* < 0.001) groups. Moreover, the EWG group exhibited higher body weight than LWG (MD: 7.92 kg; *p* < 0.001).

Likewise, the initial (W_(104.118, 2)_ = 10771; *p* < 0.001) and gestational (W_(104.505, 2)_ = 29002; *p* < 0.001) BMI differed between the groups. The initial BMI of the AWG group is lower than the LWG (MD: -3.60 kg/m^2^; *p* < 0.001) and EWG (MD: -2.47 kg/m^2^; *p* < 0.001) groups, while the BMI of the LWG group is higher in relation to the EWG group (MD: 1.13 kg/m^2^; *p* < 0.001).

For gestational BMI, the difference between the AWG group and the LWG group reduces (MD: -1.41 kg/m^2^; *p* < 0.001), while the difference between the AWG and EWG group increases (MD: -4.41 kg/m^2^; *p* < 0.001). Correspondingly, the difference between the BMI of the LWG group and the EWG group changes (MD: -3.00 kg/m^2^; *p* < 0.001).

**Fig. 4:**
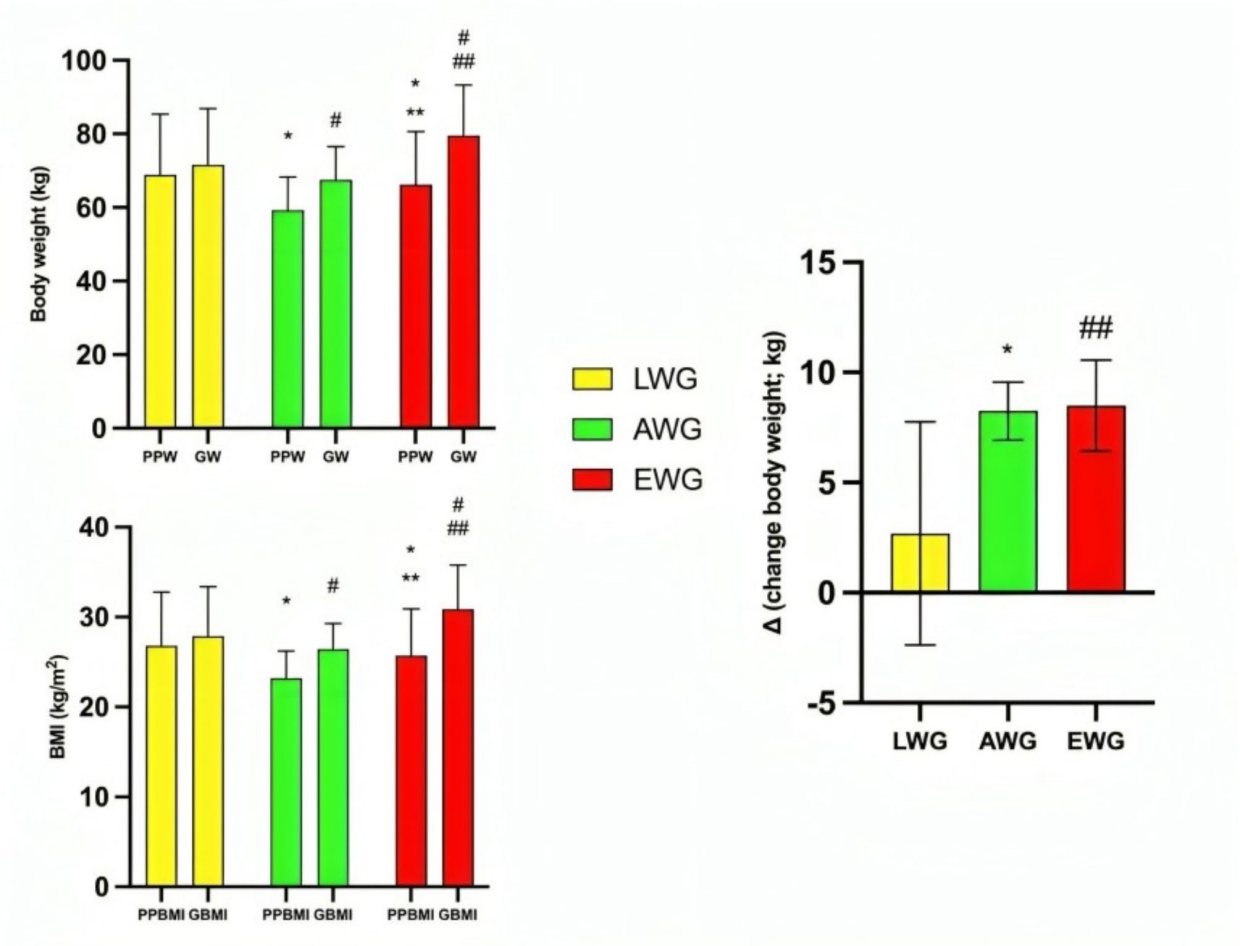
Initial and gestational body weight, BMI, and body mass change (Δ) for each group. PPW: initial body weight; GW: gestational weight; PPBMI: initial body mass index; GBMI: gestational body mass index. Analysis of variance (ANOVA) with Welch correction and Games-Howell post hoc. * different from the PPW of the LWE group; ** different from the PPW of the AWG group; # different from the GW of the LWG group; ## different from the GW group of the AWG. *p*-value ≤ 0.05

The body weight change data (final - initial; delta) between the groups also showed statistical differences (W_(127.564,118, 2)_ = 144.979; *p* < 0.001). The AWG group presented higher body mass change than the LWG group (MD: 5.56 kg; *p* < 0.001) and lower body mass change compared to the EWG group (MD: -5.06 kg; *p* < 0.001). Finally, the LWG group presents a smaller weight difference compared to the EWG group (MD: -10.62 kg; *p* < 0.001). Figure 4 illustrates initial and gestational body weight, BMI, and body mass change (Δ) for each group.

Weight gain was associated with initial BMI (X^2^_(273,569.6)_ = 13.395; *p* < 0.001), type of birth (X^2^_(273,569.2)_ = 1.183; *p* < 0.001), color/ethnicity (X^2^_(273.569, 10)_; 99.4; *p* < 0.001), marital status (X^2^_(273.569, 8)_ = 320; *p* < 0.001), women’s age (X^2^_(273.568, 2)_ = 585; *p* < 0.001) and antenatal care visits (X^2^_(273.477, 6)_ = 4.630; *p* < 0.001).

Table 2 depicts the factors associated with gestational weight gain. Underweight was associated with LWG (OR: 2.02; CI: 95% 1.89–2.15) and EWG (OR: 1.59; 95% CI: 1.49–1.68). Similarly, overweight was associated with LWG (OR: 2.07; 95% CI: 2.01–2.14) and EWG (OR: 1.74; 95% CI: 1.70–1.79). Finally, obesity was also a factor associated with LWG (OR: 7.48e + 6; 95% CI: 7.48e + 6–7.48e + 6) and EWG (OR: 5.16e + 6; 95% CI: 5.16e + 6–5.16e + 6).

**Table 2:** Factors associated with gestational weight gain during pregnancy between 2012 and 2020 in the city of São Paulo (*n* = 273.569)

	LWG vs. AWG			EWG vs. AWG		
Variable	OR	95% IC	*p*-value	OR	95% IC	*p*-value
**Maternal initial BMI** (**kg/m**^**2**^)						
18.5–24.99	1.00					
< 18.5	2.04	1.91–2.17	**< 0.001**	1.60	1.51–1.69	**< 0.001**
≥ 25.0	3.78	3.67–3.90	**< 0.001**	2.92	2.84–3.00	**< 0.001**
**Maternal age (years)**						
20–34	1.00					
≤ 15	1.10	1.009–1.20	**0.031**	0.93	0.86–1.01	0.111
16–19	1.004	0.968–1.041	0.832	0.99	0.96–1.02	0.990
≥ 35	0.98	0.94–1.021	0.353	0.82	0.79–0.84	**< 0.001**
Maternal color/ethnicity						
White	1.00					
Asians	0.76	0.63–0.92	**0.005**	0.84	0.72–0.98	**0.030**
Indigenous	0.95	0.75–1.22	0.959	0.80	0.64–0.99	**0.044**
Brown	1.00	0.97–1.03	0.717	1.04	0.98–1.02	0.743
Black	1.09	1.04–1.15	**< 0.001**	0.98	0.94–1.02	0.395
**Marital status**						
Married	1.00					
Divorced	0.87	0.77–0.98	**0.033**	0.91	0.82–1.01	0.108
Single	0.96	0.93–1.00	0.068	0.88	0.85–0.91	**< 0.001**
Stable union	0.97	0.93–1.01	0.137	0.92	0.89–0.95	**< 0.001**
**Schooling level**						
High School	1.00					
No education	0.83	0.55–1.24	0.374	0.95	0.68–1.34	0.789
Basic school 1	0.97	0.88–1.06	0.504	0.97	0.90–1.05	0.487
Basic school 2	0.99	0.95–1.02	0.571	0.99	0.96–1.02	0.696
Incomplete College	1.04	0.98–1.11	0.153	1.01	0.95–1.06	0.702
College	0.95	0.90–1.01	0.113	0.94	0.90–0.99	**0.036**
**Number of antenatal care visits**						
≥ 7	1.00					
0	1.81	1.18–2.77	**0.006**	0.72	0.48–1.09	0.125
1–3	2.21	2.02–2.43	**< 0.001**	0.58	0.53–0.63	**< 0.001**
4–6	1.33	1.29–1.38	**< 0.001**	0.64	0.62–0.66	**< 0.001**

kg: kg/m^2^: kilograms per square meter; n = number of observations in absolute values; % proportion of the number of observations; Multinominal logistic regression analysis. Outcome: LWG vs. AWG and EWG vs. AWG. The alpha error adopted to reject the null hypothesis was 5%

Women aged < 15 years showed higher odds for LWG (OR: 1.10; 95% CI: 1.00–1.20), while those aged 35–49 years reduced the odds for LWG (OR: 0.93; 95% CI: 0.89–0.97) and EWG (OR: 0.80; 95% CI: 0.78–0.83). Age > 49 years also decreased the odds of LWG (OR: 2.53e-7; 95% CI: 2.53e-7–2.53e-7). Considering ethnicity, Asian women showed lower odds for LWG (OR: 0.78; 95% CI: 0.64–0.94), while Black women present higher odds for LWG (OR: 1.06; 95% CI: 1.01–1.11). Moreover, a stable union reduced the odds of EWG (OR: 1.06; 95% CI: 1.01–1.11). Considering schooling level, we observed that basic school 2 (OR: 1.08; 95% CI: 1.05–1.12) and college (OR: 1.08; 95% CI: 1.01–1.15) increased the odds for LWG. Moreover, basic school 1 (OR: 0.82; 95% CI: 0.76–0.89), basic school 2 (OR: 0.91; 95% CI: 0.88–0.94) decreased the odds for EWG. In contrast, incomplete college (OR: 1.09; 95% CI: 1.02–1.14) and complete college (OR: 1.08; 95% CI: 1.02–1.14) increased the odds for EWG. Finally, the number of antenatal care visits was associated with GWG. For instance, considering 7 visits or more as reference, none (OR: 1.89; 95% CI: 1.23–2.90), from one to three (OR: 2.22; 95% CI: 2.02–2.44) and from four to six (OR: 1.32; 95% CI: 1.29–1.39) visits increased the odds for LWG. From one to three (OR: 0.59; 95% CI: 10.54–0.65) and from four to six (OR: 0.65; 95% CI: 10.62–0.67) decreased the odds for EWG.

## Discussion

We aimed to evaluate GWG, and associated factors of pregnant women monitored at SUS in the city of São Paulo between 2012 and 2020. Our data revealed that 86.4% of the sample showed inadequate GWG, with 21.9% LGW and 64.5% EGW. The main factors associated with LWG were obesity, overweight, underweight, age (< 15 years, 35–49 years and > 49 years), color/ethnicity (Asian and Afro-descendants) and number of consultations. The factors related to EGW were obesity, overweight and underweight, age (35–49 years), and marital status (single and stable union).

The studies mainly use the IOM guidelines to verify the GWG. However, the INTERGROWTH-21st [12] standards may be more generalizable to women in low- or middle-income countries’ settings than the IOM guidelines [4]. We applied the recent Kac et al. [13]charts to define GWG to avoid underestimations in the first trimester. The GWG is critical and is considered an essential indicator for monitoring maternal and fetal health. Previous studies suggest that LWG increases the chance of having a small gestational age neonate [17].

Like our findings, previous studies showed that initial underweight increased the odds of LWG, and initial overweight or obesity increased the odds of EWG [18]. Contrary to our findings, studies suggest that Asia was categorized as having GWG below the guidelines [19]. Our findings revealed that Asian women had lower odds of LWG compared to white women.

Interestingly, we found that more antenatal care visits were associated with GWG. For instance, compared with seven visits or more, all categories (none, from one to three and from four to six antenatal care visits) increased the odds for LWG. Likewise, from one to three and four to six antenatal care visits decreased the odds of EWG.

In Brazil, the Ministry of Health suggests at least six appointments with doctors, nurses, dentists, and other health professionals. The consequences of inadequate weight gain during pregnancy range from an increased risk of preeclampsia, gestational diabetes, complications during childbirth, postpartum weight retention, and chronic diseases to complications for the newborn, such as higher odds for preterm birth, infant mortality, alterations in the child’s body composition, and non-communicable chronic diseases in adulthood [20–23].

EWG is the primary nutritional problem to be addressed in prenatal care provided by primary health care services in the city of São Paulo. Living with overweight and obesity increases the odds of EWG. Therefore, avoiding overweight and obesity before pregnancy is essential. Previous studies showed that overweight and obesity during pregnancy are critical factors for health-related problems for mothers and newborns [24, 25].

The worldwide incidence and prevalence of overweight and obesity have increased substantially over the past few decades. This fact is consistent with the current situation in Brazil [26]. Likewise, women are more likely to become obese in the coming years [26]. Studies show that access to nutritional care in health services contributes to improved diet quality and appropriate GWG for women, especially those who are overweight or obese [27, 28]. Hence, encouraging the action of nutritionists and other health professionals who contribute to weight management is essential in health services that operate with women during the gestational period.

In our study, LWG, AWG, and EWG women were mainly in high school, being 64.1, 66.8, and 67.9%, respectively. We found that basic school 1 and college increased the odds for LWG, while basic school 1 and basic school 2 decreased the odds for EWG. Likewise, the incomplete college and college increased the odds for EWG. It is believed that poor schooling levels usually have low incomes as well, which contributes to several gestation-related problems. Furthermore, a higher educational level may promote healthier food choices and help prevent excessive weight gain during pregnancy [29–31]. Moreover, in Brazil, Black women are subjugated, and racism is still a complex factor. Racism and racial discrimination against Afro-Brazilians remain a major social and political problem in Brazil [32]. Black mothers may `have less access to health services and even less access to healthy food, factors that may favor the inadequate weight gain observed in the country. Previous data showed that experiencing racial discrimination led to an increase in obesity and worsening dietary practices, leading to more significant consumption of ultra-processed food [33].

One of this study’s notable strengths is its novel approach (using Kac et al. guidelines). The information from the SIGA database, which has never been analyzed before, provides a renewed perspective, as the only study that was done before using the SIGA database was related to data quality [14], highlighting the importance of better collection and recording of information. Furthermore, the absence of previous studies analyzing the nutritional status of pregnant women in the city of São Paulo counts for the originality of this research. The GWG studies are critical because they directly influence the development of public policies. They assist health-related managers in making informed decisions about resource allocation, enhancing prenatal care, monitoring GWG, and providing crucial nutritional guidance for this population. Thus, we strongly advocate for further studies on the dietary patterns of SUS users to identify dietary factors related to GWG.

Our study presents some limitations, such as the use of administrative data (data obtained in the work routine of health professionals) rather than research-related data for prenatal monitoring, which can lead to inconsistent measures and records. However, it is known that health professionals in the city of São Paulo’s health units were trained in anthropometry, which may minimize measurement and recording errors. Another limitation was the evaluation of pregnant women up to 40 weeks. Kac et al. charts only support pregnant women from 10 to 40 weeks. And finally, this study was not controlled for gestational diseases such as diabetes and gestational hypertension.

## Conclusions

The proportion of women with inadequate weight gain (low and excessive) is high, relating initial BMI, type of birth, color/ethnicity, marital status, women’s age and antenatal care visits. Among women with LWG, the largest proportion was of brown women, aged 20–34, who had an adequate weight at the beginning of pregnancy, who had a vaginal delivery, and who attended 7 or more antenatal care visits. Among women with EWG, the largest proportion was of women aged 20–34, single, who had a vaginal delivery, and who also had an adequate weight at the beginning of pregnancy.

Our results emphasized the significance of body weight control both before and during pregnancy. Interventions such as nutritional education in primary care and prenatal care should be suggested to help achieve adequate GWG and encourage more people to study weight gain in local data, as well as improve data quality.

## Electronic supplementary material

Below is the link to the electronic supplementary material.

Supplementary Material 1

## Data Availability

Availability of data and materialsThe full dataset and technical appendix are available from thedata custodian (Centro Universitário São Camilo) 29-10-2024-banco-inglês.xlsx . The presented data are anonymised, and risk of identification of individual participants is low.

## Abbreviations

GWG: Gestational weight gain
BMI: Body mass index
LWG: Low weight gain
AWG: Adequate weight gain
EWG: Excessive weight gain
SIGA: Primary Health Information System
SINASC: Live Birth Information System
ANOVA: Analysis of variance
