# Supplementary material for: Nutritional status and factors associated with gestational weight gain in the city of São Paulo, 2012 to 2020: a retrospective cohort study
Source: BMC Pregnancy Childbirth. 2024 Nov 13;24:746. doi: 10.1186/s12884-024-06955-5 (PMC11562861; doi:10.1186/s12884-024-06955-5)
Supplement: Supplementary file 1 — Supplementary Material 1 [file 12884_2024_6955_MOESM1_ESM.docx]

**Supplementary Material 1**

Description of the variables used in the study.

| **Variable** | **Type** | **Description** |
| --- | --- | --- |
| **Mother's height** | Original | Mother's height on the date of first care (cm). |
| **Mother's date of birth used to calculate maternal age** | Original | Mother's date of birth. |
| **Type of pregnancy** | Original | Single/ Double/ Triple or more. |
| **Number of Antenatal care visits** | Original | None / 1 to 6 visits / 7 or more. |
| **Gestacional weeks** | Original | Gestational age (in weeks) at which the visit occurred. |
| **Type of birth** | Original | Cesarean/Vaginal. |
| **Weight on date of service** | Original | Mother's weight on the date of care (kg). |
| **Race/color of skin** | Original | White /Black / Yellow / Brown/ Indigenous. |
| **Marital status** | Original | Stable union (marriage not officialized by the government)/ Divorced / Widow |
| **Schooling level** | Original | Without / Basic studies 1 /Basic studies 2/ High school /Incomplete college / Complete college |
| **Initial wejght** | Created | First weight measured prenatally, as long as it occurred before 13 weeks of gestation. |
| **Final weight** | Created | Last weight measured during prenatal care, as long as it occurred within 15 days before birth. |
| **Initial gestational age** | Created | Gestational age at the start of prenatal care, as long as it occurred before 13 weeks of gestation. |
| **Final gestational age** | Created | Gestational age at the last prenatal consultation, as long as this took place up to 15 days before birth. |
| **Initial BMI** | Created | Calculated using the formula (weight(kg)/height(m)^2^). Considering initial weight and height. |
| **Final BMI** | Created | Calculated using the formula (weight(kg)/height(m)^2^). Considering final weight and height. |
